# Selenium nanovirus and its cytotoxicity in selenite-exposed higher living organisms

**DOI:** 10.1016/j.bbrep.2020.100733

**Published:** 2020-01-25

**Authors:** Peng Bao, Guo-Xiang Li, Yu-Qin He, Hong-Yun Ren

**Affiliations:** a Key Lab of Urban Environment and Health, Institute of Urban Environment, Chinese Academy of Sciences, Xiamen, 361021, China; b Ningbo Urban Environment Observation and Station, Chinese Academy of Sciences, Ningbo, 315800, China; c University of Chinese Academy of Sciences, Beijing, 100049, China

**Keywords:** Selenite, Nanovirus, Entropy, Environmental toxicology, Cancer

## Abstract

Selenium (Se) is an essential micronutrient in living organisms, having a narrow margin between essential and potentially toxic intake/exposure. Thus, the biochemistry of Se in living organisms must be studied in-depth to determine the underlying mechanism of Se cytotoxicity. In this study, we report the emergence of selenium nanovirus (SeNVs) in selenite-exposed fish (freshwater and saltwater) and plants (dryland) and its toxicity in them. SeNVs were found in both the abdomen and tail of *Oryzias melastigma* and saltwater *Rhodeus ocellatus*, which led to their death. The occurrence of the intracellular assembly of SeNVs was observed in the roots and leaves of corn *Zea mays*, but not in those of *Limnobium laevigatum*. SeNVs led to the death of *Z. mays* but caused chronic toxicity in *L. laevigatum*. SeNVs should be a system or structure that dissipates the intracellular redox gradients of the host cells, with simple information consisting Se–O, Se–N, or Se–S bond, that would ensure elemental Se ligand binding with nearly specific biomolecules in host cells, thereby maintaining their composition and stabilizing their structure. The multiple toxic effects of Se, therefore, could be the consequence of increase of entropy in the host cells caused by the intracellular assembly of SeNVs. This study may provide an insight into the underlying mechanism of Se in environmental toxicology and its applications in human health.

## Highlights

• Intracellularly assembly of SeNPs has been considered as a new interpretation of selenium cytotoxicity.
• We provide the concept of SeNVs, which should be a system/structure that dissipates the redox gradients of host cells.
• The toxic effect of selenium could be the consequence of structural and functional disorder in the host cell caused by SeNVs.
• The concept of SeNVs will be beneficial for understanding the toxicology of selenium.

## 1 Introduction

Selenium (Se) is a micronutrient present in living organisms, essential for their normal growth and development apart from plants [[1], [2], [3], [4], [5]]. However, excessive amounts of Se can be very toxic because the margin between essential intake/exposure and potential toxicity is narrow [[6], [7], [8], [9]]. The cytotoxicity of Se has long been considered to be related to oxidative stress[10], i.e., Se species, particularly selenite and selenate, react with thiols and generate oxygen free radicals that are toxic to cells [11,12]. However, the underlying mechanism of Se cytotoxicity requires an in-depth study of the biochemistry of Se in living organisms.

The intracellular assembly of SeNPs have been found in bacteria, archaea, and fungi [[13], [14], [15], [16], [17], [18]]. The acute cytotoxicity of Se was recently attributed to the intracellular assembly of SeNPs in cancer cell lines and murine H22 hepatocarcinoma mouse model [[19], [20], [21]]. This process was deemed as the detoxification process of Se with features common to those of Se reduction into elemental Se, which bond with proteins of the host cells for self-assembly of SeNPs, but produce backfired toxicity (Schematic diagram) [14,17]. A kinetic study of the intracellular SeNPs showed that it was possibly pumped out of the microbial cells [15] or disassembled in mature cancer cells [19], which closely fits the definition of a virus, i.e., no translation, division, or energy production [22,23].

In the present study, our operational hypothesis was that the intracellular biogenic SeNPs are nanovirus (SeNVs) and are a process rather than a live system [24]. Although SeNVs have no nucleic acids, they may have a very simple clay genetic coding system [[25], [26], [27]], such that through the formation of Se–O, Se–N, or Se–S bond with proteins, their composition is maintained and stabilized [28,29]. SeNVs might be slightly toxic to the microbes that transport them out of cells, but might be highly toxic to higher living organisms [19,20]. In our previous studies, the sequestration of glycolytic enzymes by SeNVs dramatically inhibited ATP generation, which led to the functional and structural disruption of mitochondria. The sequestration of insoluble tubulin led to microtubule depolymerization, altering microtubule dynamics. In addition, the surface activity of SeNVs generated oxidative stress, thereby contributing to Se cytotoxicity. We revealed that the multiple mechanisms of Se-induced cytotoxicity were caused by SeNVs and suggested that they could potentially be the primary cause of such a type of cytotoxicity [19,20]. Except for human cancer cells, SeNVs have not yet been found in the tissues of higher organisms. In this study, we evaluated the emergence of SeNVs in selenite-exposed fish (freshwater and saltwater) and plants (dryland and aquatic) and the toxicity of SeNVs in these models.

## 2 Results and discussion

### 2.1 TEM analyses of the intracellular assembly of SeNVs in medaka, Chinese bitterling, corn, and smooth frogbit

**Fig. 1:**
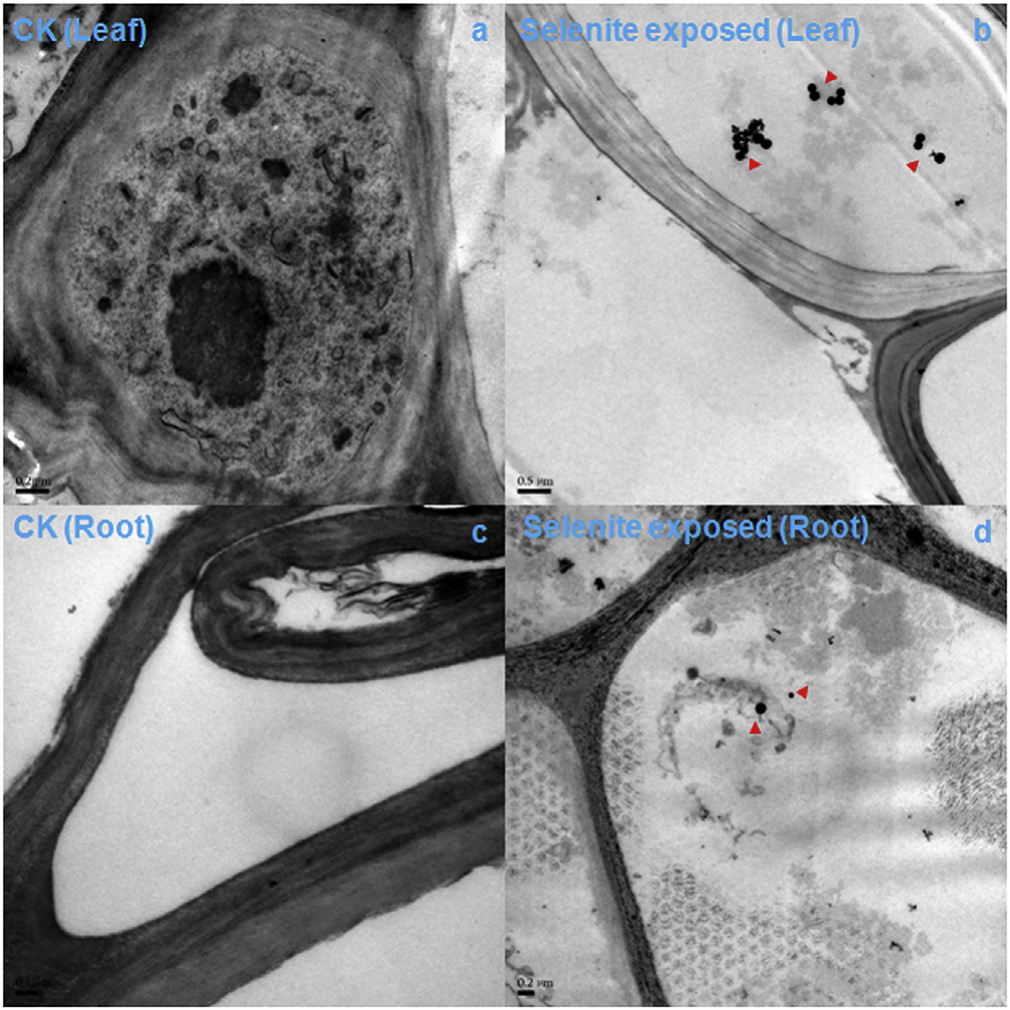
TEM images of corn (*Zea mays*) tissues, control leaf (a), selenite-exposed leaf (b), control root (c), and selenite-exposed root (d). Red triangles point to SeNVs on image.

The results of TEM analyses showed that exposure to high concentrations of selenite led to acute toxicity in medaka, Chinese bitterling, and corn with 100% mortality occurring after 24 and 96 h (Table 1). A high level of selenite exposure led to chronic cytotoxicity in smooth frogbit (Fig. S1), perhaps due to its strong methylation potential. In land plants, the absorption of selenate is mediated by a sulfate transporter [30]. A few land plants have been found to have up to 1000-fold of Se accumulation[31], which has led to the elucidation of the metabolic pathway of Se, including Se accumulation and methylation [32]. Land plants can metabolize inorganic Se to non-toxic organic compounds, such as Se-methylselenocysteine and γ-glutamyl-Se-methylselenocysteine, to reduce the toxic effects of Se [32]. However, only a few studies have reported on the selenite transporter in wheat and the green alga *Chlamydomonas reinhardtii* that revealed no requirement for Se [33,34]. In this study, significant intracellular self-assembly of SeNVs was observed in corn *Z. mays* (Figs. 1 and S2a). Corn *Z. mays* may use phosphorus transporter for selenite transport. This result may imply that there is selenite transporter in corn *Z. mays*. Therefore, the acute cytotoxicity of selenite in corn *Z. mays* may be due to the intracellular self-assembly of SeNVs (Schematic diagram) [20].

**Table 1:** Mortality of medaka, Chinese bitterling, corn and smooth frogbit after exposure to selenite.

Medaka Chinese bitterling		Corn Smooth frogbit	
time (h) mortality (%) time (h) mortality (%)		time (h) mortality (%) time (h) mortality (%)	
24	100 24 100	24	0 24 0
48	100 48 100	48	0 48 0
72	100 72 100	72	0 72 0
96	100 96 100	96	100 96 0

**Fig. 2:**
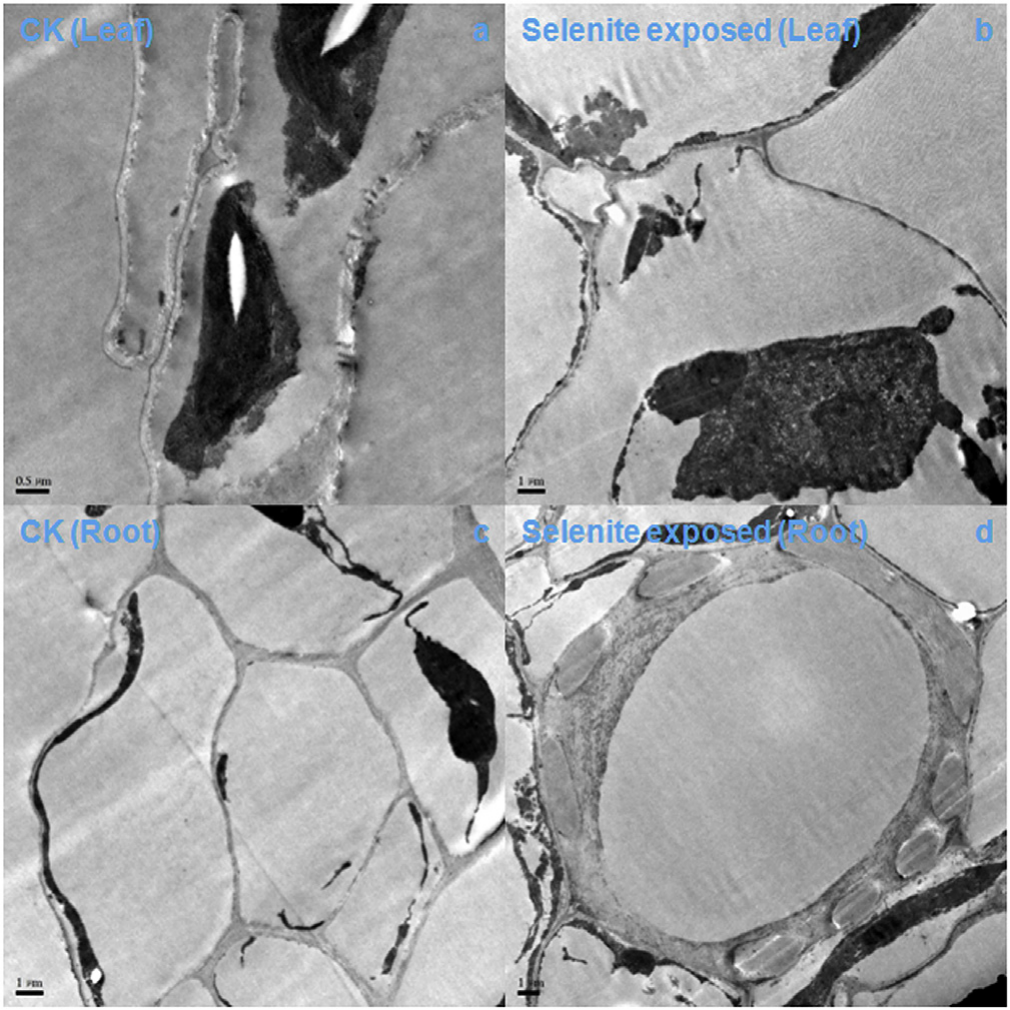
TEM images of smooth frogbit (*Limnobium Laevigatum*) tissues, control leaf (a), selenite-exposed leaf (b), control root (c), and selenite-exposed root (d).

Aquatic organisms tend to possess more Se-containing proteins than terrestrial organisms [35]. This may be because the utilization of Se is easier in aquatic environments than in terrestrial environments; thus, Se-containing proteins may have been lost during evolution from aquatic to terrestrial habitats. We did not detect Se-containing proteins or methyl selenium in smooth frogbit *L. laevigatum*, but found different toxicities between smooth frogbit *L. laevigatum* and corn *Z. mays*. Smooth frogbit *L. laevigatum* was more resistant to selenite than corn *Z. mays*, perhaps because there is no SeNVs emergence in smooth frogbit *L. laevigatum* (Fig. 1, Fig. 2). Indeed, SeNVs were not found in the leaves or roots of smooth frogbit *L. laevigatum* (Fig. 2).

**Fig. 3:**
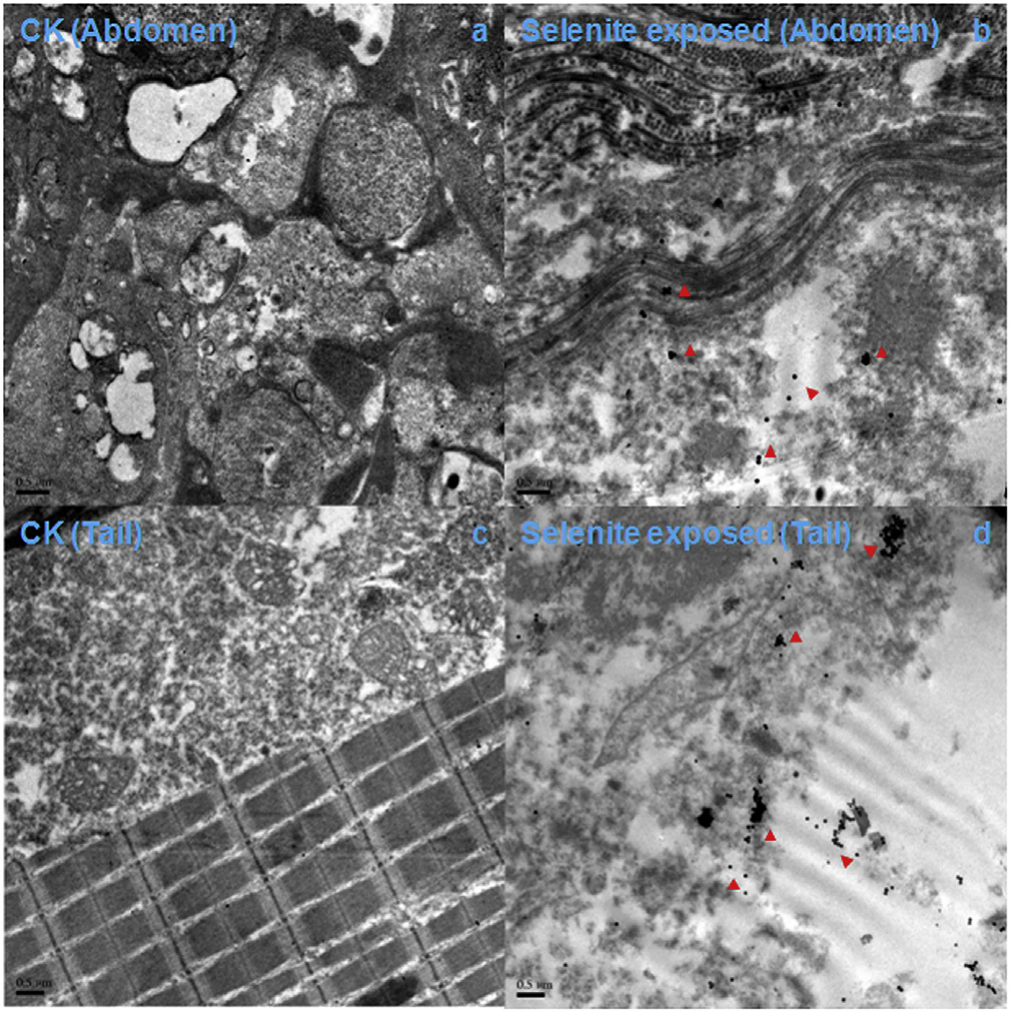
TEM images of medaka (*Oryzias melastigma*) tissues, control abdomen (a), selenite-exposed abdomen (b), control tail (c), and selenite-exposed tail (d). Red triangles point to SeNVs on image.

**Fig. 4:**
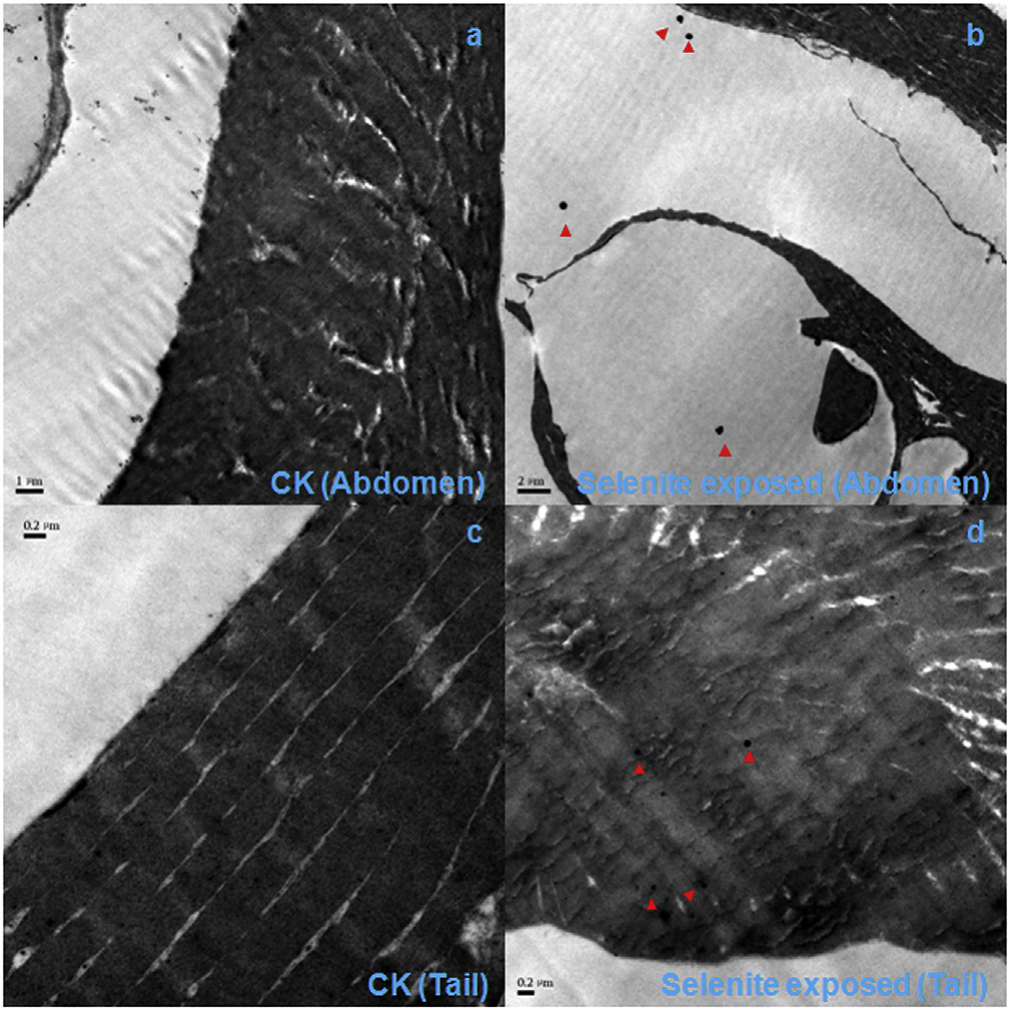
TEM images of Chinese bitterling (*Rhodeus ocellatus*) tissues, control abdomen (a), selenite-exposed abdomen (b), control tail (c), and selenite-exposed tail (d). Red triangles point to SeNVs on image.

Fig. 3, Fig. 4 show SeNVs emergence in the abdomen and tail of both selenite-exposed groups of medaka *O. melastigma* and Chinese bitterling *R. ocellatus* (Figs. S2b and c). The underline mechanism of SeNP distribution characteristics in fish might be that selenite was absorbed by the abdomen, and leading to the formation of SeNP in the abdomen. A portion of selenite was transferred by blood to the active tissues, such as tails and muscle, where selenite was reduced and SeNVs assembled. SeNVs led to the death of both medaka *O. melastigma* and Chinese bitterling *R. ocellatus* after 24 h, implying that selenite uptake and SeNVs assembly were not different in saltwater and freshwater fish. We have not yet evaluated SeNVs assembly *in vivo* using other types of Se. However, there is evidence that long-term administration of as little as 200 μg per day of selenomethionine or selenized yeast is associated with an increased incidence of alopecia, dermatitis, and type 2 diabetes [36]. Thus, SeNVs might emerge in other higher organisms when they are exposed to other forms of Se. For technical reasons, we have not determined the dynamic assembly and disassembly of SeNVs in the tissues of higher organisms that were first found in selenite-exposed H157 cancer cells [19].

### 2.1 Definition of SeNVs

In this study, we concentrated more on defining SeNVs and determining if they are living organisms. More than hundreds of definitions of life exist today [37,38] and are more often than not in conflict with one another [39]. Biologists and physicists have their own definitions. According to the summary by Trifonov, life is a metabolizing material information system with the ability to evolve and reproduce, which requires energy and a suitable environment. There are other definitions of life as follows: “*A chemical entity that consists of bounded microenvironments in chemical disequilibrium with their environment, capable of maintaining a low entropy state by energy and environment transformation, and capable of information encoding and transfer*” [40] and “*Life is a far from equilibrium self-maintaining chemical system capable of processing, transforming and accumulating information acquired from the environment*” [41]. From these points of view, SeNVs are not alive but a material system that require energy and a suitable environment.

When physicists think about the question “What is life?” the concepts of entropy and free energy play central roles. We were inspired by the concept that life should be recognized as an emerging structure that can dissipate entropy from the core of our planet, i.e., geochemical redox gradients [42]. The intracellular assembly of SeNVs requires the reducing power of host cells [13,14]. From this point of view, SeNVs should be a system or structure that dissipates the intracellular redox gradients of host cells. Host cells could be considered a microcosm that provides free energy and materials for the intracellular assembly of SeNVs. On the other hand, SeNVs dissipate energy and outputs entropy to host cells in order to maintain its structure. The multiple toxic effects of Se, therefore, could be the consequence of increase of entropy in the host cells caused by the intracellular assembly of SeNVs. In particular, if the dynamic assembly and disassembly of SeNVs occur in host cells/tissues, the metabolic disorder of matter and energy are exacerbated.

**Scheme 1:**
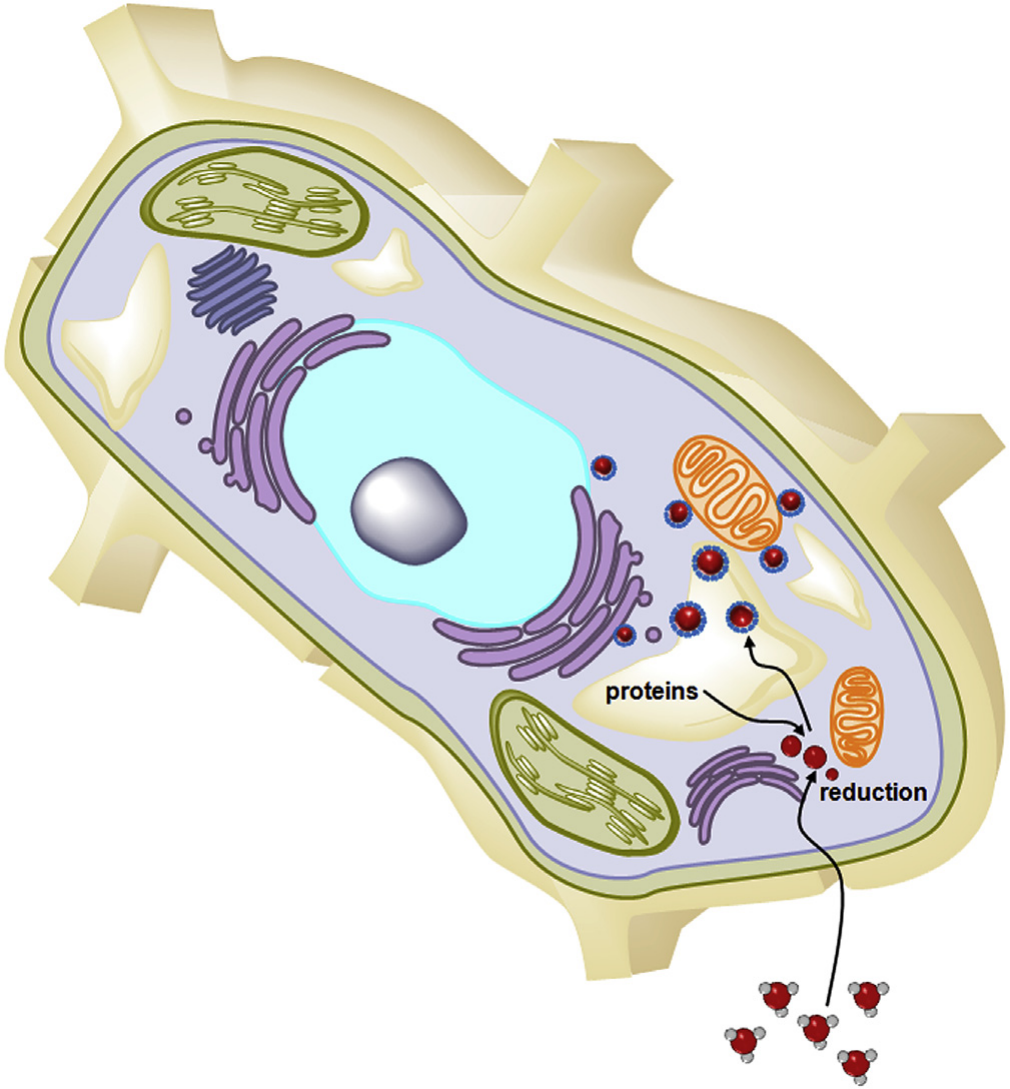
Illustration of intracellular assembly of SeNVs in selenite-exposed cells. selenite; elemental selenium; proteins; SeNVs.

From a genetic point of view, although SeNVs have no nucleic acids, they may have a very simple information storage system, through which Se–O, Se–N, or Se–S bonding to host cell biomolecules enable energy use and structure formation (Scheme 1) [29]. The simple information consisting Se–O, Se–N, or Se–S bond ensures elemental Se ligand binding with nearly specific biomolecules in host cells, thereby maintaining and stabilizing the composition of SeNVs.

## 3 Conclusion and implications

A large number of studies have shown that selenite-induced cytotoxicity is dependent on its *in vivo* speciation, giving rise to reactive oxygen species generation, autophagy inhibition, protein synthesis impairment, DNA strand breaks, microtubule depolymerization, and cell proliferation inhibition [36]. However, only the intracellular assembly of SeNPs can explain the multiple mechanisms of selenite-induced cytotoxicity, and the margin between essentiality and potential toxicity is narrow [13,14]. In this study, we developed a theory for the intracellular assembly of SeNPs and introduced the concept of SeNVs to thoroughly understand the environmental toxicology of Se. To the best of our knowledge, this study presents the first definitions of SeNVs as a system or structure that dissipates the intracellular redox gradients of host cells.

In particular, our results might provide an explanation for the astounding gap between the efficacy observed in laboratory studies and the mixed results of clinical trials for cancer therapy. Se metabolic speciation in *in vivo* laboratory and clinical studies may explain some of this efficacy gap. In other words, the intracellular assembly of SeNVs might play a central role in cancer therapy that is similar to oncolytic viruses that selectively infect and damage cancerous tissues, causing less harm to normal tissues [43]. Selenite has been shown to exhibit greater toxicity toward cancerous than normal cells[44,45],indicating its oncolytic viral role in cancer therapy—the more intracellular SeNVs, the better the efficacy. Therefore, individual differences and the choice of Se supplement speciation should be considered in clinical cancer therapy to generate more intracellular SeNVs in cancerous tissues.

This simple study might provide an insight into Se toxicity. First, we may need to re-think the mechanism of environmental toxicology of Se and the corresponding environmental policies. Second, there may be metallic- and/or alloy-based nanoviruses that can cause environmental toxicity, such as tellurium and Cu–Se. Third, an effort should be made to re-evaluate the efficacy and administration of Se in clinical cancer therapy, especially for selenite. Fourth, developing special artificial materials that facilitate intracellular self-assembly of nanoviruses may precede traditional oncolytic virus strategy in clinical cancer therapy.

## 4 Materials and methods

### 4.1 Materials and experimental design

The freshwater quality was controlled as follows: pH, 7.0–7.6 and water temperature, 25–30 °C. The brackish water quality was controlled as follows: pH, 7.0–7.6; water temperature, 25–30 °C; and salinity, 26‰.

Smooth frogbit (*Limnobium laevigatum*) was cultured in 5.0-L aquaria with fresh water, using the same freshwater quality and selenite treatment employed for the Chinese bitterling. Selenite was diluted with a stock solution (10 mg/mL), and was randomly administered to the plants (8 per group) to the final concentration of 10 mg/L. Corn (*Zea mays*) was cultivated in pots during the kharif season in 2017 in the net house of Ningbo Urban Environment Observation and Research Station, Chinese Academy of Sciences. The soil used in the pot for the experiment was collected from an agricultural farm, air-dried, ground, and sieved through a 2-mm sieve. The sieved soil (0.5 kg) was used to fill each pot. The experimental design was laid out in triplicate. Each pot had two baby corn plants and were treated with selenite (10 mg/kg) to the final concentration of 10 mg/kg after 20 days of cultivation. A batch of adult salinity-tolerant medaka (*Oryzias melastigma*) and freshwater Chinese bitterling (*Rhodeus ocellatus*) were used in this study. The fish were cultured in 5.0-L aquaria. All fish were fed an artificial pellet diet twice a day. For acute toxicity study, three separate trials were performed using the same batch. Sodium selenite (10 mg/mL) was randomly administered to adult fish (8 per group) to the final concentration of 10 mg/L [46]. Selenite was diluted with a stock solution using dechlorinated water and administered to the experimental fish. The mortality of the fish was assessed every 24 h after exposure to selenite.

### 4.2 Sample preparation and transmission electron microscopy (TEM) measurement

Tissue samples for TEM (Hitachi H-7500, Hitachi Ltd., Tokyo, Japan) were first washed with phosphate-buffered saline solution, fixed overnight *in situ* with 4% glutaraldehyde, and buffered with 0.1 M phosphate (pH 7.4) at 4 °C. The tissue samples were then fixed in 1% osmium tetroxide buffered with veronal for 1 h. The samples were then dehydrated in an alcohol series (10 min each in 25%, 50%, 75%, 85%, 95%, and 100% ethanol twice) and embedded in Epon 812 (SPI-CHEM, USA). Ultrathin sections were cut perpendicular to the plane of the monolayer and double stained with lead citrate and uranyl acetate. Electron microscopy and measurement of precipitated Se with electron microscopy-based energy dispersive X-ray (EDX) spectrometric analysis were conducted using TEM equipped with scanning and point EDX capability.

## Declaration of competing interest

The authors declare no competing interests.

## Author statement

The data used to support the findings of this study are included within the article and the supplementary information file.
