# Supplementary material for: Selenium nanovirus and its cytotoxicity in selenite-exposed higher living organisms
Source: Biochem Biophys Rep. 2020 Jan 25;21:100733. doi: 10.1016/j.bbrep.2020.100733 (PMC6992533; doi:10.1016/j.bbrep.2020.100733)
Supplement: Multimedia component 1 [file mmc1.docx]

**Supplementary Material**

Selenium nanovirus and its cytotoxicity in selenite-exposed higher living organisms

Peng Bao^1, 2, *^, Guo-Xiang Li^1,^ ^2, 3^, Yu-Qin He^1,^ ^2, 3^, Hong-Yun Ren^1^

^1^ Key Lab of Urban Environment and Health, Institute of Urban Environment, Chinese Academy of Sciences, Xiamen 361021, People’s Republic of China

^2^ Ningbo Urban Environment Observation and Station, Chinese Academy of Sciences, Ningbo 315800, People’s Republic of China

^3^ University of Chinese Academy of Sciences, Beijing 100049, People’s Republic of China

Address correspondence to:

Dr. Peng Bao

Institute of Urban Environment, Chinese Academy of Sciences, Xiamen 361021, People’s Republic of China

E-mail: [pbao@iue.ac.cn](mailto:pbao@iue.ac.cn)

Figure S1. Morphology of smooth frogbit, ck and selenite-exposed treatment.

Figure S2. Energy dispersive X-ray spectroscopy (EDX) spectrum of the electron-dense of SeNVs in corn (a), medaka (b) and Chinese bitterling (c).
